# Generalized Synchronization with Uncertain Parameters of Nonlinear Dynamic System via Adaptive Control

**DOI:** 10.1155/2014/152485

**Published:** 2014-09-11

**Authors:** Cheng-Hsiung Yang, Cheng-Lin Wu

**Affiliations:** Graduate Institute of Automation and Control, National Taiwan University of Science and Technology, No. 43, Section 4, Keelung Road, Taipei 106, Taiwan

## Abstract

An adaptive control scheme is developed to study the generalized adaptive chaos synchronization with uncertain chaotic parameters behavior between two identical chaotic dynamic systems. This generalized adaptive chaos synchronization controller is designed based on Lyapunov stability theory and an analytic expression of the adaptive controller with its update laws of uncertain chaotic parameters is shown. The generalized adaptive synchronization with uncertain parameters between two identical new Lorenz-Stenflo systems is taken as three examples to show the effectiveness of the proposed method. The numerical simulations are shown to verify the results.

## 1. Introduction

The chaos synchronization phenomenon has the following feature: the trajectories of the master and the slave chaotic system are identical in spite of starting from different initial conditions or different nonlinear dynamic system. However, slight differentiations of initial conditions, for chaotic dynamical systems, will lead to completely different trajectories [1–14]. The issue may be treated as the control law design for observer of slave chaotic system using the master chaotic system so as to ensure that the controlled receiver synchronizes with the master chaotic system. Hence, the slave chaotic system completely traces the dynamics of the master chaotic system in the course of time [15–19]. The key technique of chaos synchronization for secret communication has been widely investigated. Until now, a wide variety of approaches have been proposed for control and synchronization of chaotic systems, such as adaptive control [20, 21], backstepping control [22–25], sliding mode control [26–28], and fuzzy control [29–31], just to name a few. The forenamed strategies and many other existing skills of synchronization mainly concern the chaos synchronization of two identical chaotic systems with known parameters or identical unknown parameters [32–38].

Among many kinds of chaos synchronizations, the generalized synchronization is widely studied. This means that there exists a given functional relationship between the states of the master system and that of the slave system *y* = *f*(*x*). In this paper, a new generalized synchronization with uncertain parameters,

(1) x˙=f(t,x,A(t)),y=x+F(t),

is studied, where *x*, *y* are the state vectors of the master and slave system, respectively, and the *A*(*t*) is uncertain chaotic parameters in *f*. The *F*(*t*) may be given a regular/chaotic dynamical system.

The rest of the paper is organized as follows. In Section 2, by the Lyapunov asymptotic stability theorem, the generalized synchronization with uncertain chaotic parameters by adaptive control scheme is given. In Section 3, various adaptive controllers and update laws are designed for the generalized synchronization with uncertain parameters of the identical Lorenz-Stenflo systems. The numerical simulation of three examples is also given in Section 3. Finally, some concluding remarks are given in Section 4.

## 2. Generalized Adaptive Synchronization with Uncertain Parameters Scheme

Consider the master system

(2) $$\begin{array}{c} \dot{x}=f\left(t,x,A\left(t\right)\right) \end{array}$$

and the slave system

(3) $$\begin{array}{c} \dot{y}=f\left(t,y,\hat{A}\left(t\right)\right)+u, \end{array}$$

where *x* = [*x*_1_,*x*_2_,…,*x*_*n*_]^*T*^ ∈ *R* ^*n*^, *y* = [*y*_1_,*y*_2_,…,*y*_*n*_]^*T*^ ∈ *R* ^*n*^ denote the master states vector and slave states vector, respectively, the *f* is nonlinear vector functions, the *A*(*t*) is uncertain chaotic parameters in *f*, the $\hat{A}(t)$ is estimates of uncertain chaotic parameters in *f*, and the *u* = [*u*_1_,*u*_2_,…,*u*_*n*_]^*T*^ ∈ *R* ^*n*^ is adaptive control vector.

Our goal is to design a controller *u*(*t*) and an adaptive law $\dot{\tilde{A}}$ so that the state vector of the slave system equation (3) asymptotically approaches the state vector of the master system equation (2) plus a given vector regular/chaotic function *F*(*t*) = [*F*_1_(*t*), *F*_2_(*t*), …, *F*_*n*_(*t*)]^*T*^, and finally the generalized adaptive synchronization with uncertain parameters will be accomplished in the sense that the limit of the states error vector *e*(*t*) = [*e*_1_, *e*_2_, …, *e*_*n*_]^*T*^ and parameters error vector $\tilde{A}(t)={\left[{\tilde{A}}_{1},{\tilde{A}}_{2},\ldots ,{\tilde{A}}_{m}\right]}^{T}$ approaches zero:

(4a) $$\begin{array}{c} \underset{t\to \infty }{\lim}e\left(t\right)=0, \end{array}$$

(4b) $$\begin{array}{c} \underset{t\to \infty }{\lim}\tilde{A}\left(t\right)=0, \end{array}$$

where *e*_*i*_ = *x*_*i*_ − *y*_*i*_ + *F*_*i*_(*t*), (*i* = 1, 2, …, *n*) and ${\tilde{A}}_{j}=A_{j}(t)-{\hat{A}}_{j}(t)$, (*j* = 1, 2, …, *m*).

From (4a), we have

(5) $$\begin{array}{c} {\dot{e}}_{i}={\dot{x}}_{i}-{\dot{y}}_{i}+{\dot{F}}_{i}\left(t\right),\;i=1,2,\ldots ,n. \end{array}$$

Introduce (2) and (3) in (5) as

(6) $$\begin{array}{c} \dot{e}=f\left(t,x,A\left(t\right)\right)-f\left(t,y,\hat{A}\left(t\right)\right)+\dot{F}\left(t\right)-u\left(t\right). \end{array}$$

A Lyapunov function candidate $V(e,\tilde{A})$ is chosen as a positive definite function as

(7) $$\begin{array}{c} V\left(e,\tilde{A}\right)=\frac{1}{2}e^{T}e+\frac{1}{2}{\tilde{A}}^{T}\tilde{A}. \end{array}$$

Its derivative along the solution of (7) is

(8) V˙(e,A~)=eT[f(t,x,A(t))−f(t,y,A^(t))+F˙(t)−u(t)]+A~TA~˙,

where *u*(*t*) and $\dot{\tilde{A}}$ are chosen so that $\dot{V}=e^{T}C_{1}e+{\tilde{A}}^{T}C_{2}\tilde{A}$, *C*_1_ and *C*_2_ are negative constants, and $\dot{V}$ is a negative definite function of *e*_1_, *e*_2_, …, *e*_*n*_ and ${\tilde{A}}_{1},{\tilde{A}}_{2},\ldots ,{\tilde{A}}_{m}$. When

(9) $$\begin{array}{c} \underset{t\to \infty }{\lim}e=0,\;\;\underset{t\to \infty }{\lim}\tilde{A}=0, \end{array}$$

the generalized adaptive synchronization with uncertain parameters is obtained.

## 3. Results of Numerical Simulation

In this section, a mathematical proof is provided for the three cases' results of numerical, adaptive synchronization, generalized adaptive synchronization, and generalized adaptive synchronization with uncertain parameters.

### 3.1. Case I Adaptive Synchronization

**Figure 1:**
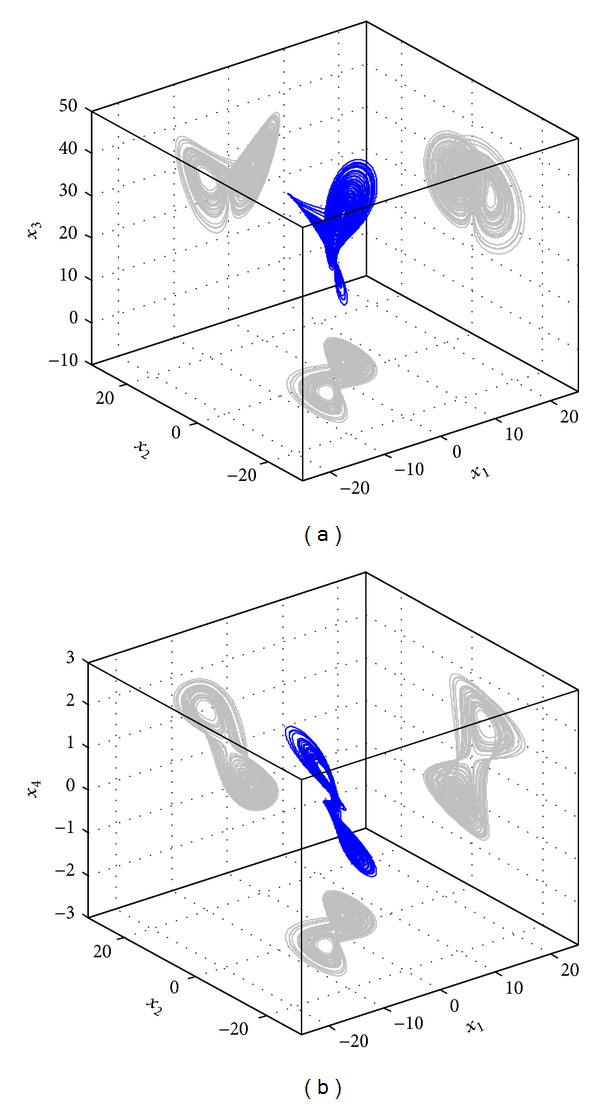
Three-dimension phase portrait of the four-dimensional Lorenz-Stenflo system and its projection.

The master system is new Lorenz-Stenflo system [39]:

(10) x˙1=a(x2−x1),x˙2=cx1−x1x3−x2,x˙3=bx4−dx3+x1x2,x˙4=−x1−ax4,

where *a* = 3.7, *b* = 1.5, *c* = 26, and *d* = 0.7. The initial conditions are *x*_1_(0) = 30, *x*_2_(0) = 30, *x*_3_(0) = 30, and *x*_4_(0) = 30. The phase portrait is shown in Figure 1.

The slave system is

(11) y˙1=a^y2−a^y1+u1,y˙2=c^y1−y1y3−y2+u2,y˙3=y1y2−d^y3+b^y4+u3,y˙4=−y1−a^y4+u4,

where $\hat{a}$, $\hat{b}$, $\hat{c}$, $\hat{d}$, and $\hat{r}$ are estimates of uncertain parameters *a*, *b*, *c*, *d*, and *r*, respectively. The initial conditions of salve system are *y*_1_(0) = −50, *y*_2_(0) = −50, *y*_3_(0) = −50, and *y*_4_(0) = −50.

Our objective is to design the controllers such that the trajectories, *x*(*t*) and *y*(*t*), of the master system and slave system satisfy

(12a) $$\begin{array}{c} \underset{t\to \infty }{\lim}||x\left(t\right)-y\left(t\right)||=0. \end{array}$$

Our objective is to design the controllers parameters estimation update laws $\dot{\tilde{A}}={\left[\dot{\tilde{a}},\dot{\tilde{b}},\dot{\tilde{c}},\dot{\tilde{d}}\right]}^{T}$ such that the trajectories, *A*(*t*) and $\hat{A}(t)$, of the uncertain chaotic parameters and estimates of uncertain chaotic parameters satisfy

(12b) $$\begin{array}{c} \underset{t\to \infty }{\lim}||A\left(t\right)-\hat{A}\left(t\right)||=0, \end{array}$$

where ||·|| denotes the Euclidean norm.

Define an error vector function

(13) $$\begin{array}{c} e_{i}=x_{i}-y_{i},\;\left(i=1,2,3,4\right). \end{array}$$

From the error functions, we get the error dynamics

(14) e˙1=a^e2+a~x2−a^e1−a~x1−u1,e˙2=c^e1+c~x1+e1e3−x1e3−x3e1−e2−u2,e˙3=−e1e2+x1e2+x2e1−d^e3−d~x3+b^e4+b~x4−u3,e˙4=−e1−a^e4−a~x4−u4,

where *e*_1_ = *x*_1_ − *y*_1_, *e*_2_ = *x*_2_ − *y*_2_, *e*_3_ = *x*_3_ − *y*_3_, *e*_4_ = *x*_4_ − *y*_4_, $\tilde{a}=a-\hat{a}$, $\tilde{b}=b-\hat{b}$, $\tilde{c}=c-\hat{c}$, and $\tilde{d}=d-\hat{d}$.

Choose a Lyapunov function candidate in the form of a positive definite function

(15) V(e1,e2,e3,e4,a~,b~,c~,d~) =12(e12+e22+e32+e42+a~2+b~2+c~2+d~2),

and its time derivative is

(16) V˙(e1,e2,e3,e4,a~,b~,c~,d~) =e1e˙1+e2e˙2+e3e˙3+e4e˙4+a~a~˙+b~b~˙+c~c~˙+d~d~˙ =e1(a^e2+a~x2−a^e1−a~x1−u1)  +e2(c^e1+c~x1+e1e3−x1e3−x3e1−e2−u2)  +e3(−e1e2+x1e2+x2e1−d^e3−d~x3+b^e4+b~x4−u3)  +e4(−e1−a^e4−a~x4−u4)+a~(−a^˙)+b~(−b^˙)  +c~(−c^˙)+d~(−d^˙)+r~(−r^˙).

Choose the parameters estimation update laws as follows:

(17) a~˙=−a^˙=−e1(x2−x1)+e4x4−a~,b~˙=−b^˙=−e3x4−b~,c~˙=−c^˙=−e2x1−c~,d~˙=−d^˙=e3x3−d~.

The initial values of estimates for uncertain parameters are $\hat{a}\left(0\right)=0$, $\hat{b}\left(0\right)=0$, $\hat{c}\left(0\right)=0$, and $\hat{d}\left(0\right)=0$. Through (16) and (17), the appropriate controllers can be designed as

(18) u1=a^e2−a^e1+e1,u2=c^e1+e1e3−x1e3−x3e1−e2+e2,u3=−e1e2+x1e2+x2e1−d^e3+b^e4+e3,u4=−e1−a^e4+e4.

Substituting (18) and (17) into (16), we obtain

(19) V˙(e1,e2,e3,e4,a~,b~,c~,d~) =−e12−e22−e32−e42−a~2−b~2−c~2−d~2<0.

**Figure 2:**
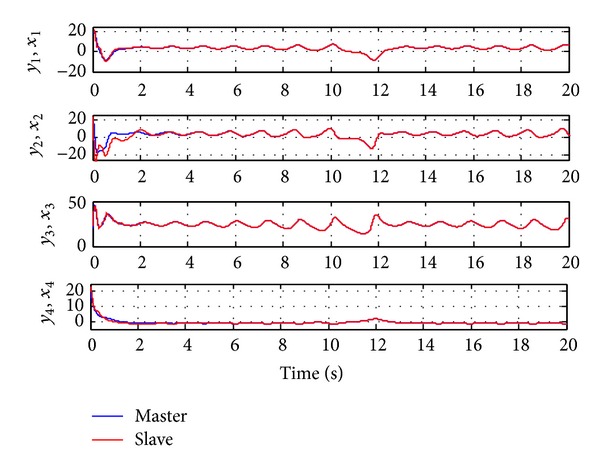
Time histories of the master system and slave system for Case I.

**Figure 3:**
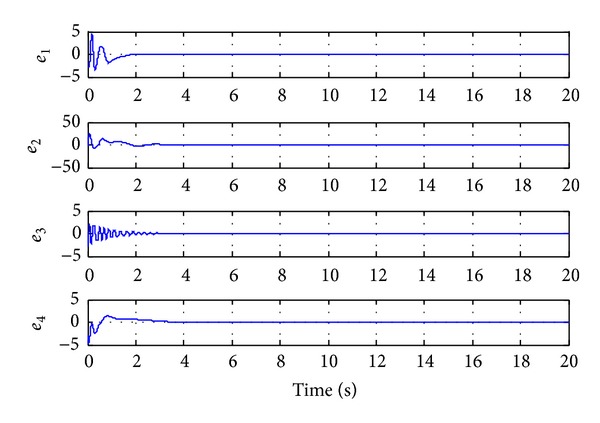
Time histories of error states for Case I.

**Figure 4:**
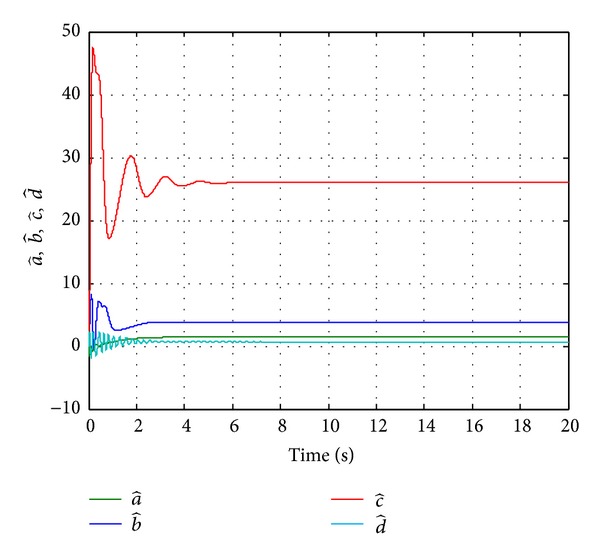
Time histories of estimated parameters for Case I.

Since the Lyapunov function $V(e_{1},e_{2},e_{3},e_{4},\tilde{a},\tilde{b},\tilde{c},\tilde{d})$ is positive definite and its derivative $\dot{V}(e_{1},e_{2},e_{3},e_{4},\tilde{a},\tilde{b},\tilde{c},\tilde{d})$ is negative definite in the neighborhood of the zero solutions for (12a) and (12b), according to the Lyapunov stability theory, the zero solutions of error states dynamic and parameters error vector are asymptotically stable; namely, the slave system equation (11) can asymptotically converge to its master system equation (10) with the adaptive control law equation (18) and the estimation parameter update law equation (17). The adaptive synchronization concept proof had to be completed. The numerical simulation results are shown in Figures 2, 3, and 4.

### 3.2. Case II Generalized Adaptive Synchronization

The given functional system for generalized synchronization is also a new Lorenz-Stenflo system but with different initial conditions: *w*_1_(0) = 25, *w*_2_(0) = 25, *w*_3_(0) = 25, and *w*_4_(0) = 25:

(20) w˙1=a(w2−w1),w˙2=cw1−w1w3−w2,w˙3=bw4−dw3+w1w2,w˙4=−aw4−w1.

When the time approaches infinite, the error functions approach zero. The generalized adaptive synchronization can be accomplished as

(21) $$\begin{array}{c} \underset{t\to \infty }{\lim}e=0, \end{array}$$

where the error functions here can be defined as

(22) $$\begin{array}{c} e_{i}=x_{i}+w_{i}-y_{i},\;\left(i=1,2,3,4\right). \end{array}$$

From the error functions equation (22), we get the error dynamics

(23) e˙1=a^(e2−e1)+a~(x2−x1+w2−w1)−u1,e˙2=c^e1+c~(x1+w1)−e2+x1(w3−e3)+w1(x3−e3)−e1(x3+w3−e3)−u2,e˙3=−d^e3−d~(x3+w3)+b^e4+b~(x4+w4)−x1(w2−e2)−w1(x2−e2)+(x2+w2−e2)−u3,e˙4=−e1−a^e4−a~(x4+w4)−u4,

where $\tilde{a}=a-\hat{a}$, $\tilde{b}=b-\hat{b}$, $\tilde{c}=c-\hat{c}$, and $\tilde{d}=d-\hat{d}$.

Choose a Lyapunov function in the form of a positive definite function

(24) V(e1,e2,e3,e4,a~,b~,c~,d~) =12(e12+e22+e32+e42+a~2+b~2+c~2+d~2),

and its time derivative is

(25) V˙(e1,e2,e3,e4,a~,b~,c~,d~) =e1e˙1+e2e˙2+e3e˙3+e4e˙4+a~a~˙+b~b~˙+c~c~˙+d~d~˙ =e1(a^(e2−e1)+a~(x2−x1+w2−w1)−u1)  +e2(c^e1+c~(x1+w1)−e2+x1(w3−e3)+ w1(x3−e3)−e1(x3+w3−e3)−u2)  +e3(−d^e3−d~(x3+w3)+b^e4+b~(x4+w4)−x1(w2−e2)−w1(x2−e2) +e1(x2+w2−e2)−u3)  +e4(−e1−a^e4−a~(x4+w4)−u4)+a~(−a^˙)+b~(−b^˙)  +c~(−c^˙)+d~(−d^˙)+r~(−r^˙).

Choose the parameters estimation update laws as follows:

(26) a~˙=−a^˙=−e1(x2−x1+w2−w1)+e4(x4+w4)−a~,b~˙=−b^˙=−e3(x4+w4)−b~,c~˙=−c^˙=−e2(x1+w1)−c~,d~˙=−d^˙=e3(x3+w3)−d~.

The initial values of estimates for uncertain parameters are $\hat{a}\left(0\right)=0$, $\hat{b}\left(0\right)=0$, $\hat{c}\left(0\right)=0$, and $\hat{d}\left(0\right)=0$. Through (25) and (26), the appropriate controllers can be designed as

(27) u1=a^e2−a^e1+e1,u2=c^e1−e2+x1(w3−e3)+w1(x3−e3)−e1(x3+w3−e3)+e2,u3=−d^e3+b^e4−x1(w2−e2)−w1(x2−w2)+e1(x2+w2−e2)+e3,u4=−e1−a^e4+e4.

Substituting (26) and (27) into (25), we obtain

(28) V˙(e1,e2,e3,e4,a~,b~,c~,d~) =−e12−e22−e32−e42−a~2−b~2−c~2−d~2<0.

**Figure 5:**
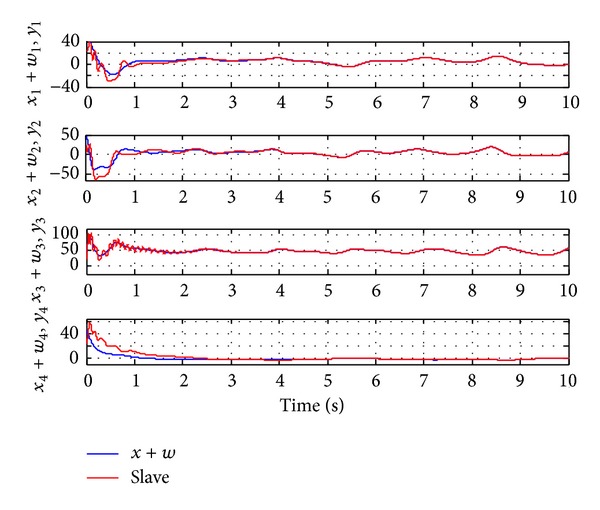
Time histories of the generalized signal (master *x* system plus given function *w*) and slave system for Case II.

**Figure 6:**
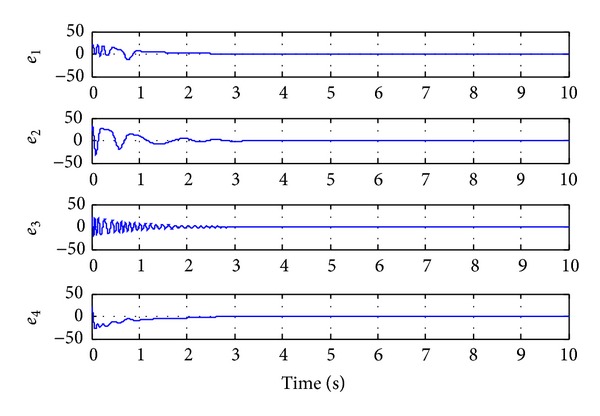
Time histories of error states for Case II.

**Figure 7:**
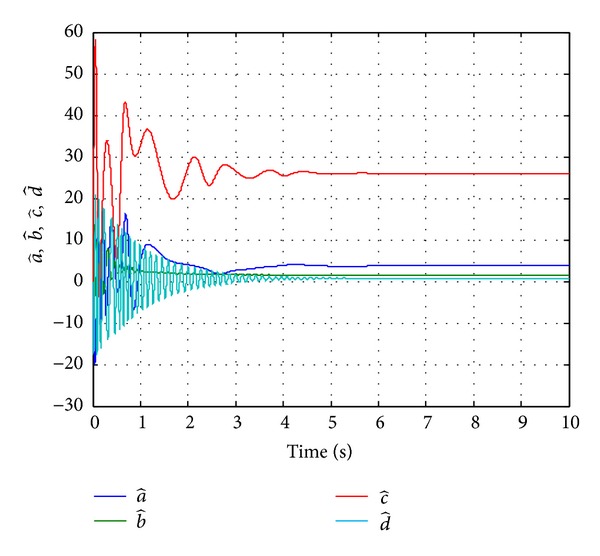
Time histories of estimated parameters for Case II.

Since the Lyapunov function $V(e_{1},e_{2},e_{3},e_{4},\tilde{a},\tilde{b},\tilde{c},\tilde{d})$ is positive definite and its derivative $\dot{V}(e_{1},e_{2},e_{3},e_{4},\tilde{a},\tilde{b},\tilde{c},\tilde{d})$ is negative definite in the neighborhood of the zero solutions for (12a) and (12b), according to the Lyapunov stability theory, the zero solutions of error states dynamic and parameters error vector are asymptotically stable; namely, the slave system equation (11) can asymptotically converge to its master system equation (10) with the adaptive control law equation (27) and the estimation parameter update law equation (26). The generalized adaptive synchronization concept proof had to be completed. The numerical simulation results are shown in Figures 5, 6, and 7.

### 3.3. Case III Generalized Adaptive Synchronization with Uncertain Parameters

Consider that the master system is the new Lorenz-Stenflo system with uncertain chaotic parameters

(29) x˙1=A1(t)(x2−x1),x˙2=A3(t)x1−x1x3−x2,x˙3=A2(t)x3−A4(t)x3+x1x2,x˙4=−A1(t)x4−x1,

where *A*_1_(*t*), *A*_2_(*t*), *A*_3_(*t*), and *A*_4_(*t*) are uncertain chaotic parameters. The uncertain parameters are given as

(30) A1(t)=a(1+f1z1),A2(t)=b(1+f2z2),A3(t)=c(1+f3z3),A4(t)=d(1+f4z4),

where *f*_1_, *f*_2_, *f*_3_, and *f*_4_ are arbitrary positive constants. Positive constants are *f*_1_ = *f*_2_ = *f*_3_ = *f*_4_ = 0.005. The chaotic signals *z*_1_, *z*_2_, *z*_3_, and *z*_4_ are given as the states of system as follows:

(31) z˙1=a(z2−z1),z˙2=cz1−z1z3−z2,z˙3=bz4+−dz3+z1z2,z˙4=−az4−z1.

The initial constants of the chaotic signals are *z*_1_(0) = 0.7, *z*_2_(0) = 0.7, *z*_3_(0) = 0.7, and *z*_4_(0) = 0.7. The new Lorenz-Stenflo system with uncertain chaotic parameters of master system will exhibit a more complex dynamic behavior since the parameters of the system change over time.

The generalized synchronization error functions can be defined as

(32) $$\begin{array}{c} e_{i}=x_{i}+w_{i}-y_{i},\;\left(i=1,2,3,4\right). \end{array}$$

From the error functions equation (32), the error dynamics becomes

(33) e˙1=A^1(e2−e1−w2+w1)+A~1(x2−x1)+a(w2−w1)−u1,e˙2=A^3(e1−w1)+A~3x1+cw1+x1(w3−e3)+w1(x3−e3)−e1(x3+w3−e3)−e2−u2,e˙3=A^2(e4−w4)+A~2x4+bw4−A^4(e3−w3)−A~4x3−dw3−x1(w2−e2)−w1(x2−e2)+e1(x2+w2−e2)−u3,e˙4=−e1−A^1(e4−w4)−A~1x4−aw4−u4,

where ${\tilde{A}}_{i}\left(t\right)=A_{i}\left(t\right)-{\hat{A}}_{i}\left(t\right)$  (*i* = 1,2, 3,4).

Choose a Lyapunov function in the form of a positive definite function:

(34) V(e1,e2,e3,e4,A~1,A~2,A~3,A~4) =12(e12+e22+e32+e42+A~12(t)+A~22(t)+A~32(t)+A~42(t)),

and its time derivative is

(35) V˙(e1,e2,e3,e4,A~1,A~2,A~3,A~4) =e1e˙1+e2e˙2+e3e˙3+e4e˙4+A~1A~˙1+A~2A~˙2  +A~3A~˙3+A~4A~˙4 =e1(A^1(e2−e1−w2+w1)+A~1(x2−x1)+a(w2−w1)−u1)  +e2(A^3(e1−w1)+A~3x1+cw1+x1(w3−e3)+w1(x3−e3)−e1(x3+w3−e3)−e2−u2)  +e3(A^2(e4−w4)+A~2x4+bw4−A^4(e3−w3)−A~4x3−dw3−x1(w2−e2)−w1(x2−e2)+e1(x2+w2−e2)−u3)  +e4(−e1−A^1(e4−w4)−A~1x4−aw4−u4)  +A~1(−A^˙1)+A~2(−A^˙2)+A~3(−A^˙3)+A~4(−A^˙4).

Choose the parameters estimation update laws for those uncertain parameters as follows:

(36) A~˙1=A˙1−A^˙1=−e1(x2−x1)+e4x4−A~1,A~˙2=A˙2−A^˙2=−e3x4−A~2,A~˙3=A˙3−A^˙3=−e2x1−A~3,A~˙4=A˙4−A^˙4=e3x3−A~4.

The initial values of estimates for uncertain parameters are ${\hat{A}}_{1}\left(0\right)=0$, ${\hat{A}}_{2}\left(0\right)=0$, ${\hat{A}}_{3}\left(0\right)=0$, and ${\hat{A}}_{4}\left(0\right)=0$. Through (35) and (36), the appropriate controllers can be designed as

(37) u1=A^1(e2−e1−w2+w1)+a(w2−w1)+e1,u2=A^3(e1−w1)+cw1+x1(w3−e3)+w1(x3−e3)−e1(x3+w3−e3)−e2+e2,u3=A^2(e4−w4)+bw4−A^4(e3−w3)−dw3−x1(w2−e2)−w1(x2−w2)+e1(x2+w2−e2)+e3,u4=−A^1(e4−w4)−aw4−e1+e4.

Substituting (36) and (37) into (35), we obtain

(38) V˙(e1,e2,e3,e4,A~1,A~2,A~3,A~4) =−e12−e22−e32−e42−A~12(t)−A~22(t)−A~32(t)−A~42(t) <0.

**Figure 8:**
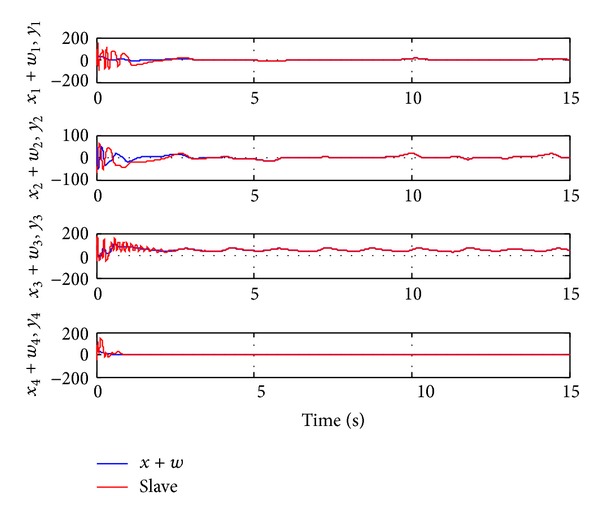
Time histories of the generalized signal (master *x* system plus given function *w*) and slave system for Case III.

**Figure 9:**
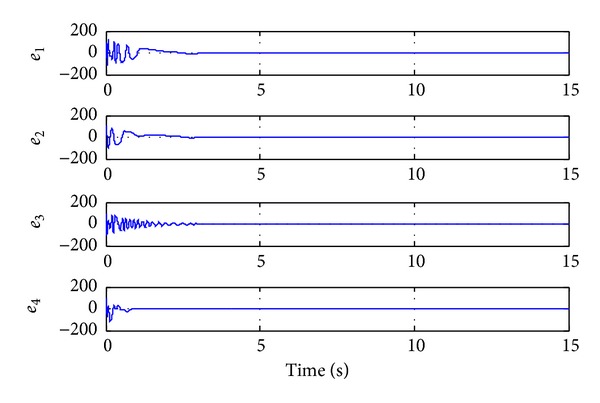
Time histories of error states for Case III.

**Figure 10:**
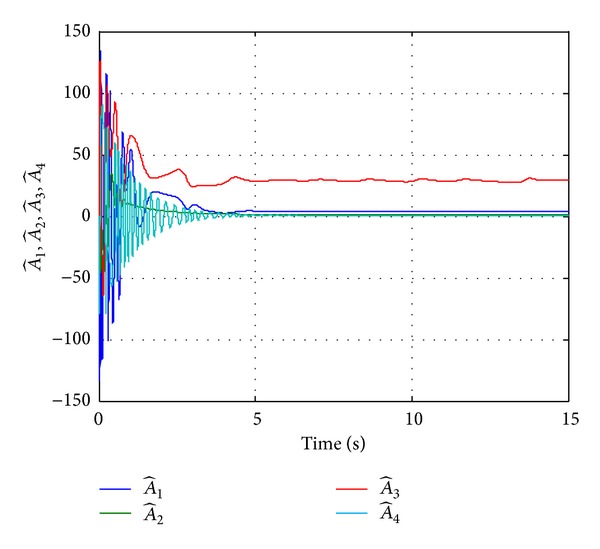
Time histories of estimated parameters for Case III.

**Figure 11:**
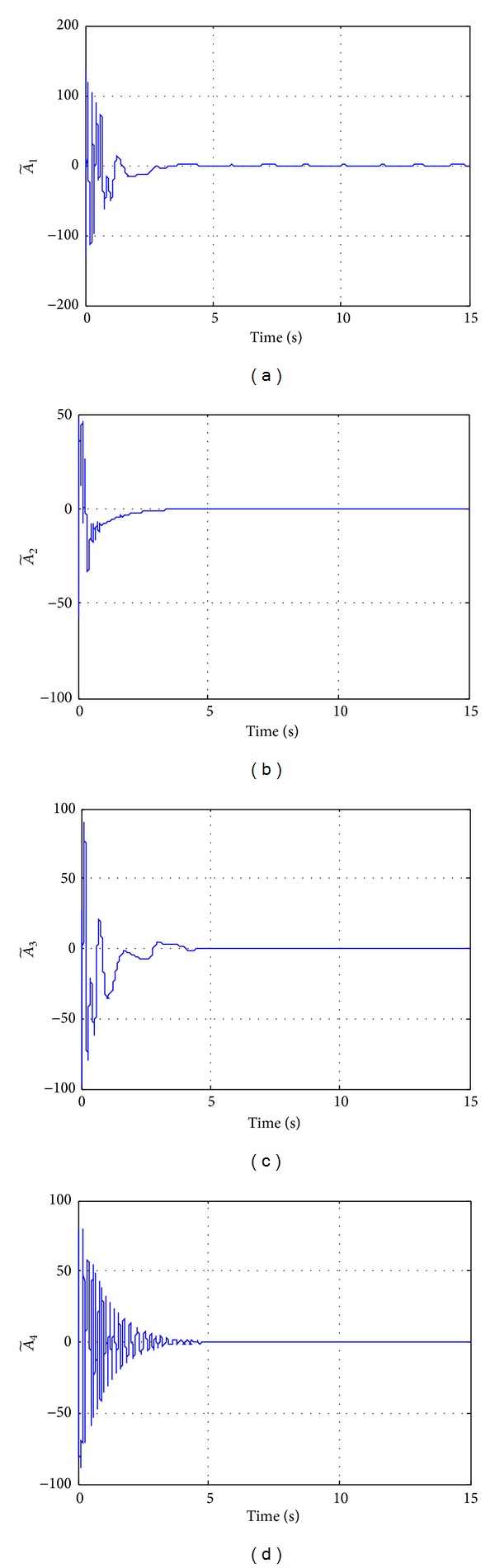
Time histories of different parameters for Case III.

Since the Lyapunov function $V(e_{1},e_{2},e_{3},e_{4},\tilde{a},\tilde{b},\tilde{c},\tilde{d})$ is positive definite and its derivative $\dot{V}(e_{1},e_{2},e_{3},e_{4},\tilde{a},\tilde{b},\tilde{c},\tilde{d})$ is negative definite in the neighborhood of the zero solutions for (12a) and (12b), according to the Lyapunov stability theory, the zero solutions of error states dynamic and parameters error vector are asymptotically stable; namely, the slave system equation (11) can asymptotically converge to its master system equation (10) with the adaptive control law equation (36) and the estimation parameter update law equation (37). The generalized adaptive synchronization with uncertain parameters concept proof had to be completed. The numerical simulation results are shown in Figures 8, 9, 10, and 11.

## 4. Conclusion

A generalized adaptive synchronization with uncertain chaotic parameters is new chaos synchronization concept. The theoretical analysis and numerical simulation results of three cases, adaptive synchronization, generalized adaptive synchronization, and generalized adaptive synchronization with uncertain parameters, are shown in the corresponding figures which imply that the adaptive controllers and update laws we designed are feasible and effective. In this paper, the three examples can be used to increase the security of secret communication system.
